# Honeycomb reactor: a promising device for streamlining aerobic oxidation under continuous-flow conditions

**DOI:** 10.3762/bjoc.19.55

**Published:** 2023-05-31

**Authors:** Masahiro Hosoya, Yusuke Saito, Yousuke Horiuchi

**Affiliations:** 1 API R&D Laboratory, Research Division, Shionogi & Co., Ltd., 1-1, Futaba-cho 3-Chome, Toyonaka, Osaka 561-0825, Japan; 2 Carbon Neutral Promotion Division, ARK Creation Centre, Cataler Corporation, 1905-10 Shimonobe, Iwata, Shizuoka 438-0112, Japan

**Keywords:** aerobic oxidation, benzaldehydes, benzyl alcohols, homogeneous catalyst, honeycomb reactor

## Abstract

We report on the high potential of a honeycomb reactor for the use in aerobic oxidation under continuous-flow conditions. The honeycomb reactor is made of porous material with narrow channels separated by porous walls allowing for high density accumulation in the reactor. This structure raised the mixing efficiency of a gas–liquid reaction system, and it effectively accelerated the aerobic oxidation of benzyl alcohols to benzaldehydes under continuous-flow conditions. This reactor is a promising device for streamlining aerobic oxidation with high process safety because it is a closed system.

## Introduction

Oxidation plays a key role in synthesizing highly functionalized molecules [1–2]. While Jones oxidation [3] and oxidation using KMnO_4_ [4] are classical and powerful methods, their harsh and hazardous conditions impede their application to complex molecules. Thus, a variety of mild and chemoselective oxidations have been developed, including Swern oxidation [5], tetrapropylammonium perruthenate (TPAP) oxidation [6], Pinnick oxidation [7], and 2,2,6,6-tetramethylpiperidinyl-1-oxyl (TEMPO) oxidation [8]. TEMPO oxidation, in particular, has been successfully applied on a manufacturing scale as a low-cost and green oxidation method [9]. However, these oxidation processes generally require stoichiometric oxidants, and reduced byproducts must be purged in a purification step, which diminishes the atom economy [2].

To overcome this limitation, the use of molecular oxygen (O_2_) present in air as an oxidant is one of the ideal solutions [10–11]. The reduction of O_2_ generates only water as a byproduct, leading to high atom-economy processes. However, the use of O_2_ as an oxidant has safety risks due to its potential for explosions when employed on a manufacturing scale [12]. These risks are due to the presence of two out of the three elements of combustion, namely combustibles, oxygen supply and an ignition source [13], and unexpected ignition caused by static electricity from operators or the equipment can have disastrous consequences. Because the large headspace of batch reactor aggravates these safety risks, the use of O_2_ in batch manufacturing is very limited [14].

Recently, continuous flow synthesis has recently been studied as a way to mitigate the safety risks [15–16]. A compact and closed system improves the process safety of handling molecular oxygen by eliminating unexpected ignition. The safety advantage stimulates the development of various aerobic oxidation processes under continuous-flow conditions accompanied by dedicated devices such as tube-in-tube reactors or fixed bed reactors [17–20].

To maximize this advantage, the gas–liquid biphasic reaction must be controlled under continuous-flow conditions. This reaction requires high mixing efficiency to assure high mass transfer of the gas to the liquid phase and a consequent high reaction rate [21]. The mechanism for mixing is categorized mainly as active and passive mixing [22]. Active mixing requires an external force and a driving part. In using O_2_, active mixing increases the risk of ignition due to friction from the driving part, making it unsuitable for the continuous system to handle aerobic oxidation. Therefore, passive mixing should be more suitable for aerobic oxidation. The whole flow reactor with passive mixing can be immersed in incombustible medium such as water, leading to the improvement of the process safety. Passive mixing is commonly realized using slug-flow [23] or a static mixer [24]. Slug-flow is a simple method for passive mixing, but the formation of slug-flow depends on the tube diameter [23], and the reaction rate decreases as the tube diameter increases [25–26]. The gas–liquid biphasic reaction also displays the same characteristic, and a static mixer needs to be developed to enable more robust aerobic oxidation under continuous-flow conditions. A static mixer is generally used by inserting it into a tube reactor. In the gas-liquid biphasic flow reaction, the static mixer has to be inserted into the full range of the tube reactor to maintain high mixing efficiency throughout the reaction. This complex equipment complicates its versatile application for the scale-up and manufacturing, which leads to higher costs.

**Figure 1:**
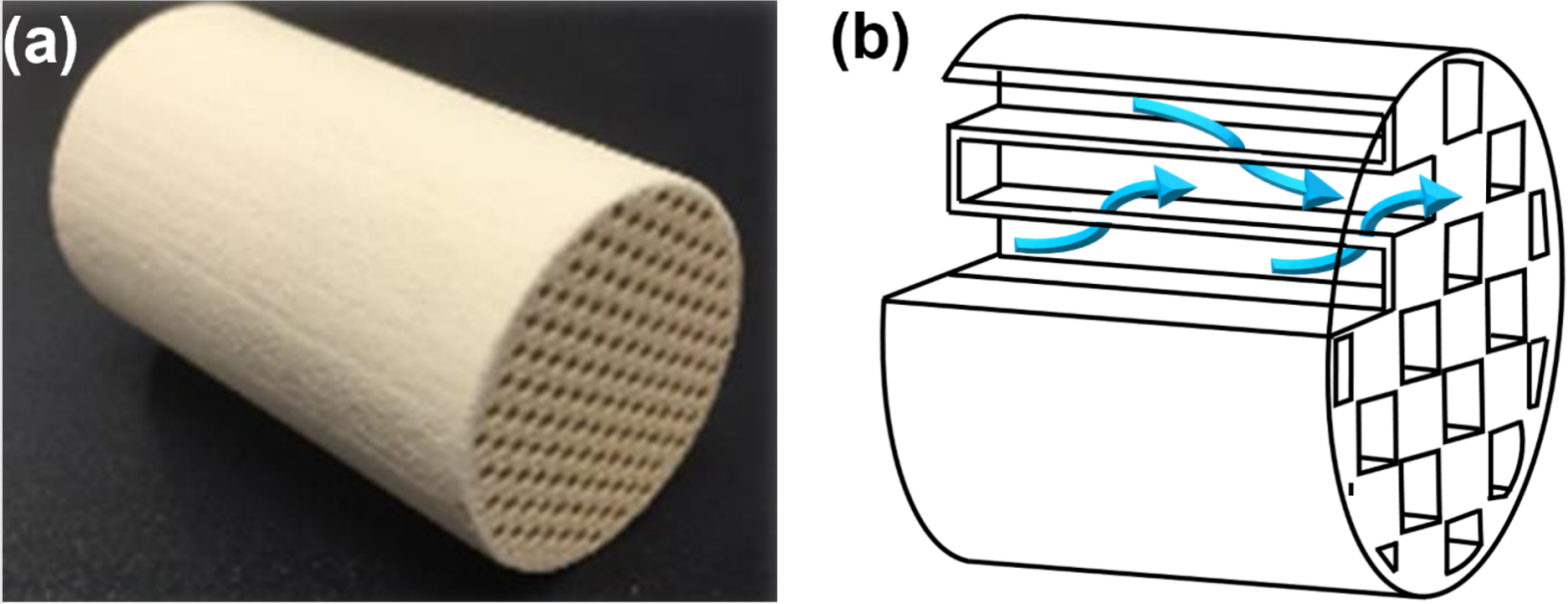
Honeycomb reactor. (a) Photograph. (b) Schematic diagram.

To address this issue, we turned our attention to the use of porous material, which is widely used for exhaust gas treatment in automobiles [27–30]. A porous device can improve gas contact efficiency in exhaust gas treatment. Cataler has developed gas–liquid mixing technology using a honeycomb reactor made of porous material (Figure 1) [31–32]. Narrow channels separated by porous walls allow for high density accumulation in the reactor. As the gas–liquid reaction mixture passes through the honeycomb reactor, the porous material functions as a static mixer, maintaining high mixing efficiency throughout the aerobic oxidation process.

We believe that this processing technology offers much potential for application as a flow reactor with a highly efficient and reliable static mixer in its full range. Such a reactor should become one of the ideal devices for aerobic oxidation under continuous-flow conditions and contribute to the further development of aerobic oxidation processes and related oxidation processes such as ozonolysis reactions [33]. Herein, we describe the feasibility of the honeycomb reactor for aerobic oxidation.

## Results and Discussion

### Reaction screening for aerobic oxidation under batch conditions

To select the representative aerobic oxidation, the reaction conditions to oxidize 4-methoxybenzyl alcohol (**1a**) were screened (Table 1). The oxidized product *p*-anisaldehyde (**2a**) is a valuable substance in food chemistry [34–35] and a valuable intermediate for synthesizing active pharmaceutical ingredients [36]. This oxidation has been often applied for evaluating catalyst activity for aerobic oxidation [37], and its screening results can be transferred to obtain a wide variety of benzaldehydes from benzyl alcohols. The screening was conducted under batch conditions. Toward its application to continuous-flow synthesis, we considered the description of the reaction mixture as well as the reaction rate, conversion, yield and availability of the catalysts. Because the honeycomb reactor is made of porous material, the homogeneous reaction solution is a key factor.

**Table 1:** Reaction screening for aerobic oxidation of 4-methoxybenzyl alcohol (**1a**).

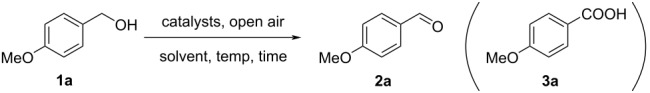									

Entry^a^	Catalysts	Solvent	Description	Temp	Time	HPLC (area %)			Conv
		
	(equiv)	(mL/g)		(°C)	(min)	**1a**	**2a**	**3a**	(%)^b^

1	TEMPO (0.05), Cu(MeCN)_4_OTf (0.05), 2,2’-bipyridyl (0.05), NMI (0.10)^c^	MeCN (12)	brown to green solution	25	30	0.0	97.7	0.4	100

2	nor-AZADO (0.01), NaNO_2_ (0.20)	AcOH (14)	pale yellow solution	25	15	8.9	90.3	0.0	42
					60	0.0	99.9	0.0	100

3	TEMPO (0.05), Fe(NO_3_)_3_·9H_2_O (0.05)	AcOH (7)	orange solution	25	60	49.3	50.7	0.0	7
					23 h	0.0	98.8	0.5	100

4	TEMPO (0.05), Cu(NO_3_)_2_·3H_2_O (0.075)	AcOH (7)	blue solution	25	60	26.9	64.2	0.0	14
					20 h	0.0	98.9	0.4	100

5	TEMPO (0.05), Zn(NO_3_)_2_·6H_2_O (0.075)	AcOH (7)	pale yellow solution	25	60	68.8	29.9	0.0	3
					20 h	0.0	98.3	0.7	100

6	PdOAc)_2_ (0.05), pyridine (0.10)	toluene (7)	pale brown slurry	50	60	24.8	72.8	0.0	17
					20 h	4.4	88.4	7.2	59

7	Cu(OAc)_2_·H_2_O (0.05), pyridine (0.60)	toluene (7)	blue slurry	50	360	97.7	1.9	0.1	0

8	Ni(OH)_2_, (0.10)	toluene (7)	green slurry	50	60	98.1	1.8	0.0	0

9	DDQ (0.10), NaNO_2_ (0.10)	AcOH (7)	black slurry	25	60	1.1	95.9	0.0	86
					360	0.0	97.0	0.0	100

10	DDQ (0.05), Fe(NO_3_)_3_·9H_2_O (0.05)	AcOH (7)	black solution	25	60	5.5	92.7	0.0	54
					360	0.6	97.9	0.1	92

^a^1.0 mmol of 4-methoxybenxyl alcohol (**1a**) was used. The reaction was conducted under open air in EYELA ChemiStation (PPS-1511) with a cross-shaped stirring bar. ^b^Conv (%) = **2a** (area %)/(**2a** (area %) + **1a** (area %) × 14.083) × 100. 14.083: relative sensitivity coefficient on HPLC (factor). ^c^NMI = *N*-methylimidazole.

Stahl and Steves have developed a highly reactive aerobic oxidation [38]. This promising methodology enables completion of the reaction in 30 min at room temperature (Table 1, entry 1). However, four kinds of catalysts were used, and a simpler catalytic system would be preferable. The highly reactive catalyst, 9-azanoradamantane *N*-oxyl (nor-AZADO), was tried with NaNO_2_ as a cocatalyst, which resulted in completion of the reaction in 60 min (Table 1, entry 2) [39]. While this led to a simpler catalyst system, nor-AZADO is expensive. Hong and co-workers have developed a low-cost catalyst system using TEMPO and nitrate salts [40]. Fe(NO_3_)_3_ (Table 1, entry 3), Cu(NO_3_)_2_ (Table 1, entry 4), Zn(NO_3_)_2_ (Table 1, entry 5) worked as catalysts combined with TEMPO. The reactivities were significantly lower than those in Table 1, entries 1 and 2, but the reaction could be completed overnight at room temperature. The catalysts were completely dissolved in AcOH and the reaction mixture remained in the solution throughout the reaction in entries 3–5. From the viewpoint of application to pharmaceutical manufacturing, the residual amount of copper must be controlled according to ICH Q3D [41]. Iron and zinc have low toxicity and are not listed in ICH Q3D. In comparison with the initial reaction rate of 60 min, Fe(NO_3_)_3_/TEMPO in Table 1, entry 3 shows high potential for further optimization. Aerobic oxidation using transition metals instead of TEMPO was also investigated. Pd(OAc)_2_ (Table 1, entry 6) [42] and Cu(OAc)_2_ (Table 1, entry 7) [43], and Ni(OH)_2_ (Table 1, entry 8) [44] left the starting material **1a**. Pd(OAc)_2_ led to moderate conversion, but Pd(OAc)_2_ did not dissolve in toluene even with pyridine. As a substitute for TEMPO, 2,3-dichloro-5,6-dicyano-*p*-benzoquinone (DDQ) was tried (Table 1, entries 9 and 10) [45]. Although the reactivity was improved compared with the TEMPO catalytic system in Table 1, entries 3–5, the DDQ catalytic system made it difficult to confirm the solubility due to the deep black color of the reaction mixture. This can increase the risk of clogging under continuous-flow conditions due to undissolved catalyst.

From this reaction screening, we concluded that Table 1, entry 3 was the most suitable catalytic system for low-cost and environmentally friendly aerobic oxidation and further optimized it to improve the reaction rate.

### Reaction optimization for aerobic oxidation under batch conditions

**Scheme 1:**
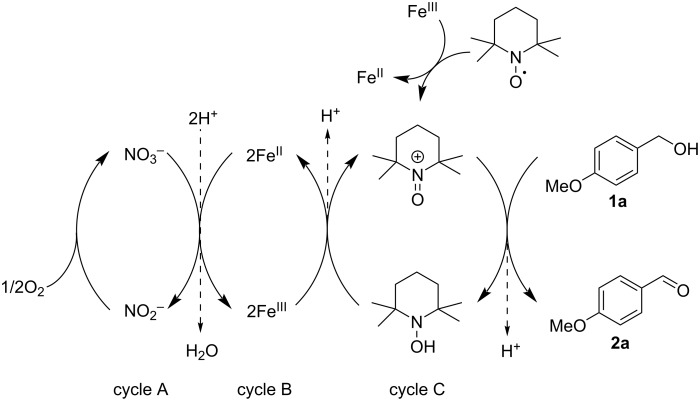
Proposed catalytic cycle for aerobic oxidation using Fe(NO_3_)_3_/TEMPO.

Based on entry 3 in Table 1, we next focused on optimizing the reaction to increase the reaction rate (Table 2). Open air was switched to an O_2_ balloon to increase the partial pressure of O_2_ (Table 2, entry 1 vs 2 and 3 vs 4). At room temperature, the reaction rate did not increase although the partial pressure of O_2_ increased approximately five times with this change. In contrast, at 60 °C, the reaction rate improved greatly with the O_2_ balloon, and the reaction was completed in 20 min. The proposed catalytic cycle for aerobic oxidation is shown in Scheme 1 [40,46–47]. At room temperature, the solubility of O_2_ in the reaction solution is relatively high, and the catalytic cycle A, which is associated with the solubility of O_2_, is not the rate-determining step. Therefore, the reaction rates under open air and O_2_ balloon did not differ. On the other hand, increasing the temperature decreased the solubility of O_2_, which converted the catalytic cycle A to the rate-determining step at 60 °C. When the amounts of catalysts were decreased to 0.02 equiv (Table 2, entry 5), the reaction completion took 60 min. When the reaction solution was heated to 80 °C, the reaction was completed within 20 min with 0.02 equiv of Fe(NO_3_)_3_/TEMPO (Table 2, entry 6). When the amounts of catalysts were decreased to 0.01 equiv at 80°C, the reaction time was significantly prolonged, which is not suitable for the flow synthesis (Table 2, entry 7). Based on these findings, entry 6 was considered to be the optimal reaction conditions, and the application to flow synthesis using the honeycomb reactor was tried under these conditions.

**Table 2:** Reaction optimization for aerobic oxidation of 4-methoxybenzyl alcohol (**1a**).

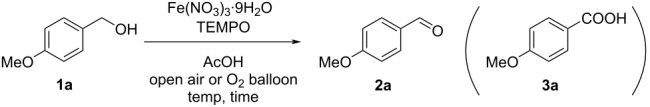									

Entry^a^	Catalysts	Solvent	Oxidant	Temp	Time	HPLC (area %)			Conv
		
	Fe(NO_3_)_3_·9H_2_O/TEMPO (equiv)	(mL/g)		(°C)	(min)	**1a**	**2a**	**3a**	(%)^b^

1	0.05/0.05	AcOH (7)	open air	25	30	65.1	34.9	0.0	4
					60	49.3	50.7	0.0	7
					150	23.8	75.6	0.0	18
					23 h	0.0	98.8	0.5	100

2	0.05/0.05	AcOH (7)	O_2_ balloon	25	60	53.9	44.6	0.0	6
					180	16.4	82.3	0.0	26

3	0.05/0.05	AcOH (7)	open air	60	10	7.5	90.9	0.0	46
					20	2.6	96.1	0.0	72
					30	0.4	98.2	0.0	95
					40	0.0	98.4	0.2	100

4	0.05/0.05	AcOH (7)	O_2_ balloon	60	10	0.5	97.5	0.0	93
					20	0.0	98.5	0.6	100

5	0.02/0.02	AcOH (3)	O_2_ balloon	60	10	33.3	62.4	0.0	12
					20	16.3	81.0	0.0	26
					30	8.5	86.5	0.0	42
					40	3.9	94.6	0.1	63
					50	0.5	98.0	0.1	93
					60	0.0	98.8	0.3	100

6	0.02/0.02	AcOH (3)	O_2_ balloon	80	10	2.8	95.3	0.1	71
					20	0.0	98.1 (≥99)^c^ (94)^d^	0.7	100

7	0.01/0.01	AcOH (1)	O_2_ balloon	80	10	20.0	76.8	0.0	21
					20	8.9	89.0	0.0	42
					30	4.2	94.0	0.1	61
					40	1.7	96.7	0.1	80
					50	0.6	97.9	0.1	92
					60	0.0	98.6	0.1	100

^a^The charge amount of 4-methoxybenxyl alcohol (**1a**): 1.0 mmol (entries 1–4), 2.5 mmol (entries 5 and 6) and 5.0 mmol (entry 7). The reaction was conducted in EYELA ChemiStation (PPS-1511) with a cross-shaped stirring bar. ^b^Conv (%) = **2a** (area %)/(**2a** (area %) + **1a** (area %) × 14.083) × 100. 14.083: relative sensitivity coefficient on HPLC (factor). ^c^Quantitative yield using an authentic sample on HPLC. ^d^Isolated yield (**1a**: 3.6 mmol scale).

### Evaluation of the heat of reaction

Prior to application to the flow synthesis, the heat of this aerobic oxidation was measured. Because oxidation is generally a highly exothermic reaction, the internal temperature distribution might vary depending on the heat removal efficiency of the flow reactors. This fluctuation makes it difficult to precisely evaluate the reaction rate.

**Figure 2:**
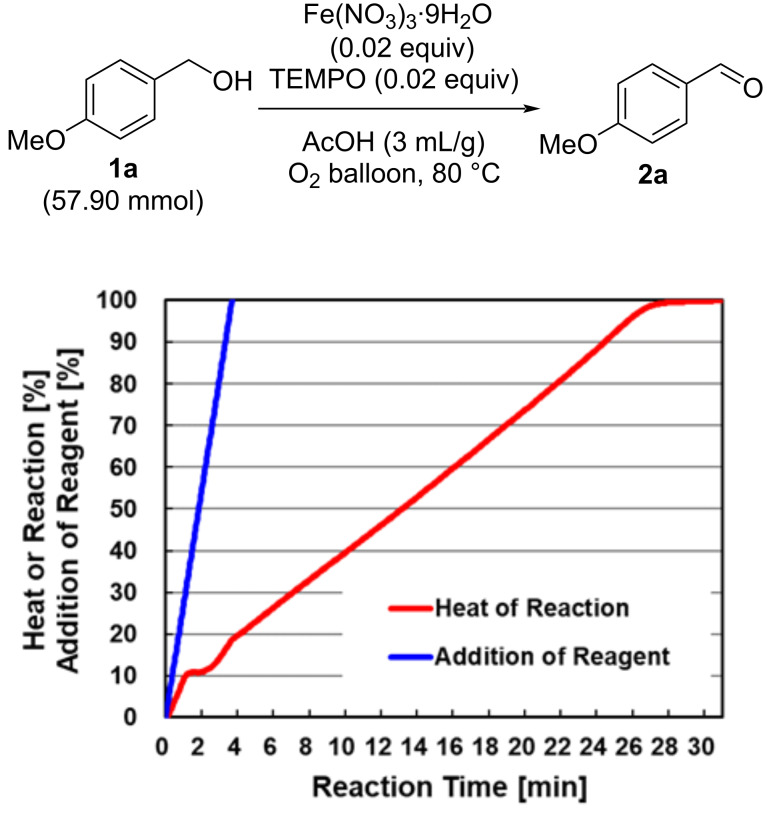
Time course of the heat of reaction for aerobic oxidation.

The heat of reaction, calculated with the heat flow during the reaction, was 161 kJ/mol (based on the input amount of the starting material **1a**, see Supporting Information File 1). The time course of the heat of reaction revealed that this reaction was almost a zero-order reaction as shown by the red line in Figure 2, which did not depend on the concentration of **1a** (Figure 2). As shown in entries 3 vs 4 in Table 2, the rate-determining step is considered to be the catalytic cycle A in Scheme 1, which is consistent with the consideration from the time course of the heat of reaction. The adiabatic heating was calculated to be 138 K (see Supporting Information File 1). To suppress the increase of the internal temperature, the amount of AcOH was increased by 40 times (from 3 mL/g to 120 mL/g) for the following evaluation of the reaction rate under flow conditions. This dilution suppressed the adiabatic heating to less than 5 K, and the reaction rate could be more precisely evaluated.

### Evaluation of the reaction rate under continuous-flow conditions

**Scheme 2:**
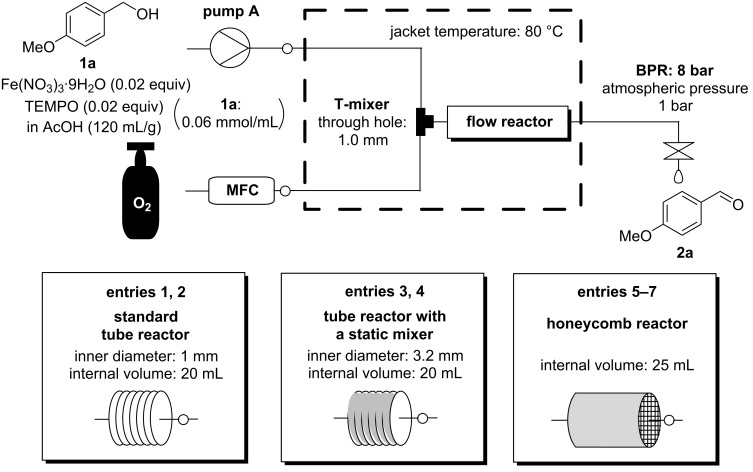
Flow setup for aerobic oxidation using various flow reactors.

**Figure 3:**
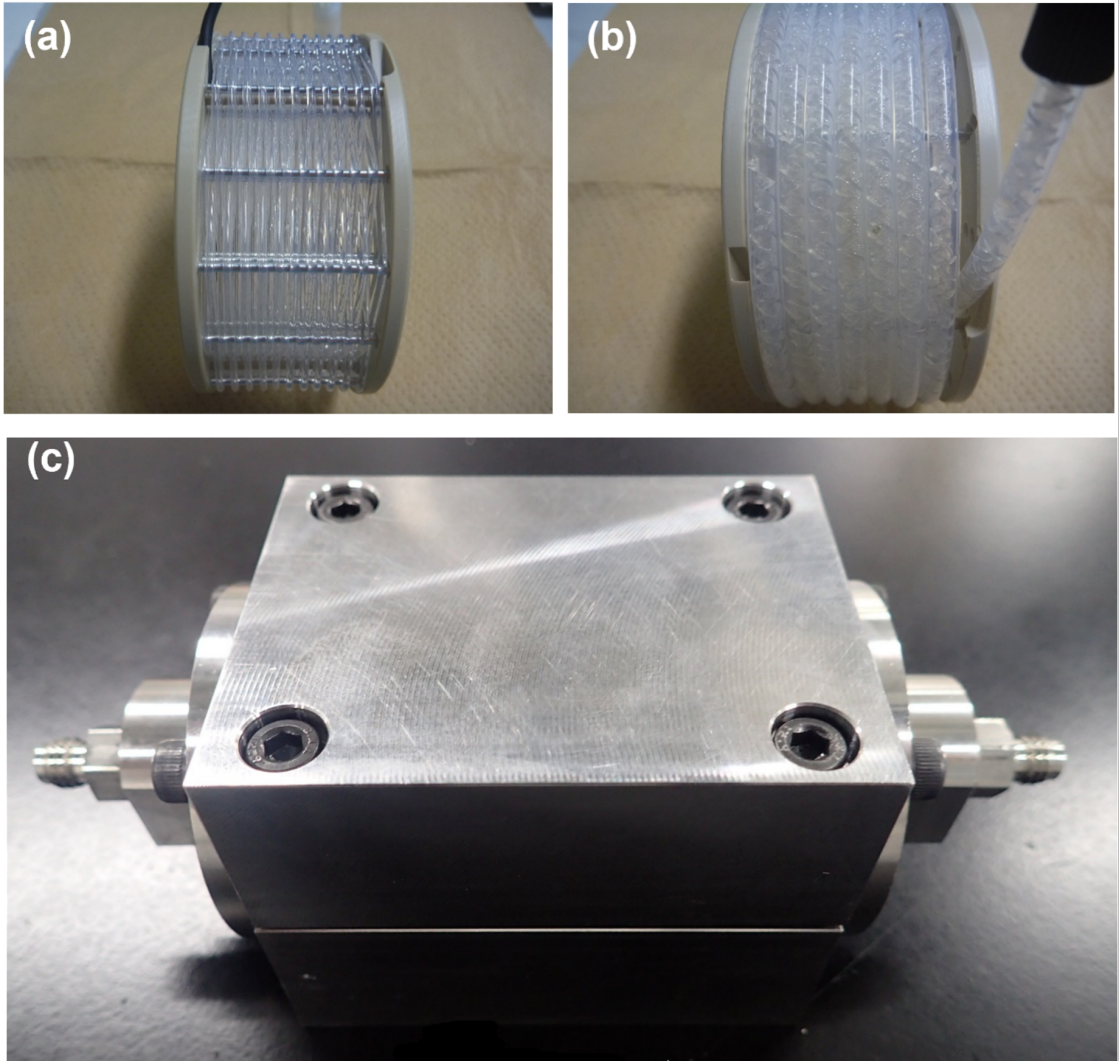
Photographs of the various reactors. (a) Standard tube reactor. (b) Tube reactor with a static mixer. (c) Honeycomb reactor.

The reaction rate was evaluated under continuous-flow conditions using a standard tube reactor, a tube reactor with a static mixer and the honeycomb reactor (Scheme 2, Figure 3 and Table 3). The ratio of flow rates to internal volumes was unified in all the reactors. In the standard tube reactor, slug-flow was formed, and the conversion was 47% (Table 3, entry 1). To evaluate the reaction rate for increasing the flow rate, the reaction solution was passed through the standard tube reactor three times (Table 3, entry 2). The internal vortex was strengthened by increasing the flow rate, and the mixing efficiency was improved to give a higher reaction rate (67%). In the tube reactor with a static mixer, the reaction rate did not depend on the flow rate although the reaction conversion was higher than that in the standard tube reactor (Table 3, entries 3 and 4). The tube reactor with a static mixer had a large inner diameter and the linear velocity became correspondingly low. The low linear velocity did not effectively affect the reaction rate. When the honeycomb reactor was set horizontally (Table 3, entry 5), the reaction conversion was almost equivalent to that in the standard tube reactor. When the honeycomb reactor was set vertically (Table 3, entry 6), the reaction rate greatly improved, and the reaction conversion was almost equivalent to that in the tube reactor with a static mixer. When the honeycomb reactor was set horizontally, oxygen gas was passed through the upper side of reactor and the reaction solution was passed through the lower side of the reactor. This heterogeneity resulted in low mixing efficiency and short pass of the reaction solution through the honeycomb reactor, which led to the low reaction rate. When the honeycomb reactor was set horizontally, the residence time distribution using the solution of **2a** in the honeycomb reactor was much shorter than expected, which supported the above consideration (see Supporting Information File 1). The reaction rate was improved in a similar manner to Table 3, entries 1 and 2 to give the highest reaction conversion (95%) when the reaction solution was passed through the honeycomb reactor three times (Table 3, entry 7). When three honeycomb reactors were connected in series (Table 3, entry 8), the reaction conversion was equivalent to that in Table 3, entry 7, which showed that three cycles in a single honeycomb reactor and one cycle in three honeycomb reactors gave the same result. Table 3, entry 8 is a more suitable flow setup for manufacturing, but Table 3, entry 7 is a more practical flow setup for laboratory experiments because the reaction conversion in each cycle can be analyzed. The porous structure in the honeycomb reactor improved the mixing efficiency like the static mixer, and the higher flow rate resulted in the higher reaction conversion. This evaluation showed the high potential of the honeycomb reactor for application to gas-liquid flow aerobic oxidation.

**Table 3:** Evaluation of the reaction rate using various flow reactors.

Entry	Pump A / MFC	Reactor	Internal volume	Cycle(s)	HPLC (area %)			Conv.
		
	(mL/min)		(mL)		**1a**	**2a**	**3a**	(%)^a^

1	0.5/8.0	standard tube	20	1	7.5	92.1	0.1	47

2	1.5/24.0	standard tube	20 (3 cycles: 60)	1	19.6	80.0	0.0	22
				2	8.6	91.0	0.1	43
				3	3.4	96.3	0.1	67

3	0.5/8.0	static mixer	20	1	1.1	98.2	0.2	86

4	1.5/24.0	static mixer	20 (3 cycles: 60)	1	16.3	83.2	0.0	27
				2	5.5	94.1	0.1	55
				3	1.2	98.1	0.2	85

5	0.63/10.0	honeycomb (horizontal)	25	1	6.7	92.3	0.4	49

6	0.63/10.0	honeycomb (vertical, upflow)	25	1	1.3	97.2	0.6	84

7	1.88/30.0	honeycomb (vertical, upflow)	25 (3 cycles: 75)	1	9.9	89.5	0.1	39
				2	1.9	97.3	0.3	78
				3	0.4	98.3	0.6	95

8	1.88/30.0	honeycomb (vertical, upflow)	25 × 3^b^	1	0.4	98.4	0.6	95

^a^Conv (%) = **2a** (area %)/(**2a** (area %) + **1a** (area %) × 14.083) × 100. 14.083: relative sensitivity coefficient on HPLC (factor). ^b^Three honeycomb reactors were connected in series.

### Maximizing the throughput of aerobic oxidation using the honeycomb reactor

**Scheme 3:**
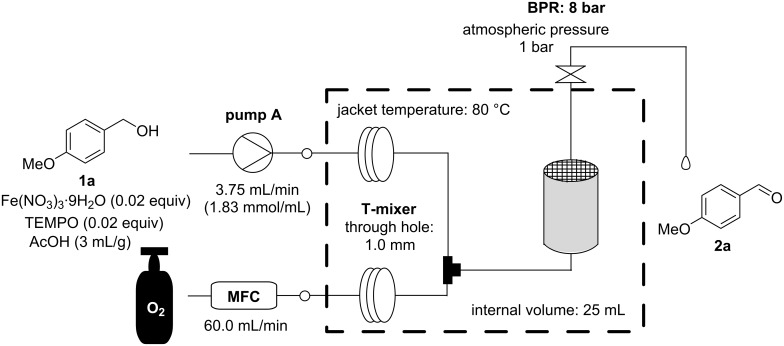
Flow setup for high-throughput aerobic oxidation using the honeycomb reactor.

Because the honeycomb reactor was found to be a suitable reactor for aerobic oxidation, the concentration of the reaction solution was returned to that of batch conditions to maximize the throughput (Scheme 3 and Table 4). When the reaction solution was passed through the honeycomb reactor three times, the reaction was completed in two cycles. The increased temperature derived from the heat of reaction accelerated the reaction. The quantitative yield by HPLC was 98.3% and the byproduct carboxylic acid **3a** was 1.3%. **3a** was easily purged by extraction with a weak base such as NaHCO_3_. From the above discussion, the throughput was improved to 1.4 kg/day (= 57 g/h = 1.83 × 3.75 × 138.17 = 948 mg/min), which is suitable for kg-scale aerobic oxidation.

**Table 4:** Reaction rate for high-throughput aerobic oxidation using the honeycomb reactor.

Pump A / MFC (mL/min)	Internal volume	Cycle(s)	HPLC (area %)			Conv.
		
	(mL)		**1a**	**2a**	**3a**	(%)^a^

3.75/60.0	25 (3 cycles: 75)	1	4.5	91.9	3.0	59
		2	0.0	95.2 (98.3)^b^	4.2 (1.3%)^b^	100
		3	0.0	94.2	5.2	100

^a^Conv (%) = **2a** (area %)/(**2a** (area %) + **1a** (area %) × 14.083) × 100. 14.083: relative sensitivity coefficient on HPLC (factor). ^b^Quantitative yield using an authentic sample on HPLC.

### Substrate scope and additional screening for aerobic oxidation using the honeycomb reactor

**Scheme 4:**
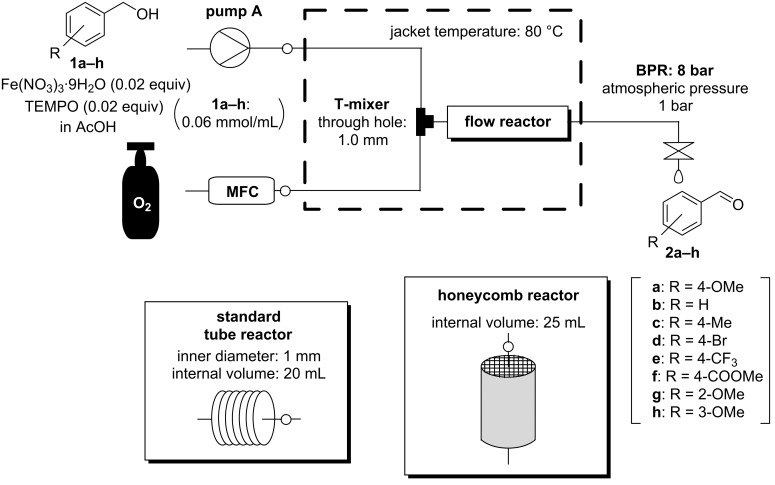
Flow setup for substrate scope and additional screening.

Various benzyl alcohols were investigated to verify the effectiveness of the honeycomb reactor. The reaction rate was compared using the standard tube reactor and the honeycomb reactor (Scheme 4 and Table 5). With benzyl alcohol and benzyl alcohols bearing various substituents such as OMe, Me, Br, CF_3_ and COOMe at the 4-position and OMe at the 2- or 3-position, the reaction was uniformly accelerated using the honeycomb reactor and proceeded almost without generation of byproducts (Table 5, entries 1–8). Although Cu(NO_3_)_2_ or Zn(NO_3_)_2_/TEMPO catalytic system worked in a similar manner to Fe(NO_3_)_3_/TEMPO catalytic system, the accelerating effect of the reaction using the honeycomb reactor was limited (Table 5, entries 9 and 10). In these catalytic systems, the solubility of O_2_ may not be the clear rate-determining step unlike Fe(NO_3_)_3_/TEMPO catalytic system. The above results indicated that the honeycomb reactor streamlines aerobic oxidation under continuous-flow conditions especially when the solubility of O_2_ is the rate-determining step.

**Table 5:** Substrate scope and additional screening for aerobic oxidation using the standard tube reactor and the honeycomb reactor.

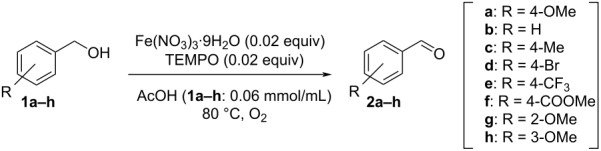				

Entry	R	Reactor	Conv.	Quantitative yield
			(%)^a^	(%)^b^

1	4-OMe	standard tube^c^	47	47
		honeycomb^d^	84	83

2	H	standard tube^c^	42	43
		honeycomb^d^	85	85

3	4-Me	standard tube^c^	39	39
		honeycomb^d^	81	79

4	4-Br	standard tube^c^	49	48
		honeycomb^d^	83	82

5	4-CF_3_	standard tube^c^	56	54
		honeycomb^d^	84	82

6	4-COOMe	standard tube^c^	64	61
		honeycomb^d^	84	81

7	2-OMe	standard tube^c^	51	51
		honeycomb^d^	74	70

8	3-OMe	standard tube^c^	61	61
		honeycomb^d^	80	79

9	4-OMe^e^	standard tube^c^	46	45
		honeycomb^d^	54	52

10	4-OMe^f^	standard tube^c^	31	32
		honeycomb^d^	44	43

^a^Conv (%) = **2a–h** (area %)/(**2a–h** (area %) + **1a–h** (area %) × F) × 100. F: relative sensitivity coefficient on HPLC (factor). Relative sensitivity coefficients were summarized in Supporting Information File 1. ^b^Quantitative yield using an authentic sample on HPLC. ^c^Reaction conditions were described in entry 1 in Table 3. ^d^Reaction conditions were described in entry 6 in Table 3. ^e^Cu(NO_3_)_2_·3H_2_O (0.03 equiv) was used instead of Fe(NO_3_)_3_·9H_2_O (0.02 equiv). ^f^Zn(NO_3_)_2_·6H_2_O (0.03 equiv) was used instead of Fe(NO_3_)_3_·9H_2_O (0.02 equiv).

## Conclusion

In aerobic oxidation of benzyl alcohols to benzaldehydes under continuous-flow conditions, using the honeycomb reactor gave a higher reaction rate than the standard tube reactor or the tube reactor with the static mixer. The honeycomb reactor is a promising device for streamlining aerobic oxidation under continuous-flow conditions with process safety [48]. The high gas–liquid contact efficiency using the honeycomb reactor can also be applied to other gas–liquid flow reaction systems.

## Supporting Information

File 1 Experimental procedures, supplementary experiments and NMR spectra.

## References

[1] Tohma H, Kita Y (2004). Adv Synth Catal.

[2] Caron S, Dugger R W, Ruggeri S G, Ragan J A, Ripin D H B (2006). Chem Rev.

[3] Bowden K, Heilbron I M, Jones E R H, Weedon B C L (1946). J Chem Soc.

[4] Shaabani A, Tavasoli‐Rad F, Lee D G (2005). Synth Commun.

[5] Mancuso A J, Huang S-L, Swern D (1978). J Org Chem.

[6] Griffith W P, Ley S V, Whitcombe G P, White A D (1987). J Chem Soc, Chem Commun.

[7] Bal B S, Childers W E, Pinnick H W (1981). Tetrahedron.

[8] Vogler T, Studer A (2008). Synthesis.

[9] Ciriminna R, Pagliaro M (2010). Org Process Res Dev.

[10] Chen K, Zhang P, Wang Y, Li H (2014). Green Chem.

[11] Liu J, Guðmundsson A, Bäckvall J-E (2021). Angew Chem, Int Ed.

[12] Cavani F, Teles J H (2009). ChemSusChem.

[13] Pekalski A A, Zevenbergen J F, Lemkowitz S M, Pasman H J (2005). Process Saf Environ Prot.

[14] Butters M, Catterick D, Craig A, Curzons A, Dale D, Gillmore A, Green S P, Marziano I, Sherlock J-P, White W (2006). Chem Rev.

[15] Gemoets H P L, Su Y, Shang M, Hessel V, Luque R, Noël T (2016). Chem Soc Rev.

[16] Hone C A, Roberge D M, Kappe C O (2017). ChemSusChem.

[17] Hone C A, Kappe C O (2019). Top Curr Chem.

[18] Wu G, Brett G L, Cao E, Constantinou A, Ellis P, Kuhn S, Hutchings G J, Bethell D, Gavriilidis A (2016). Catal Sci Technol.

[19] Durndell L J, Isaacs M A, Li C, Parlett C M A, Wilson K, Lee A F (2019). ACS Catal.

[20] Zhou W, Chen G, Yu B, Zhou J, Qian J, He M, Chen Q (2020). Appl Catal, A.

[21] Nieves-Remacha M J, Kulkarni A A, Jensen K F (2013). Ind Eng Chem Res.

[22] Hessel V, Löwe H, Schönfeld F (2005). Chem Eng Sci.

[23] Chen L, Tian Y S, Karayiannis T G (2006). Int J Heat Mass Transfer.

[24] Bertsch A, Heimgartner S, Cousseau P, Renaud P (2001). Lab Chip.

[25] Ueno M, Hisamoto H, Kitamori T, Kobayashi S (2003). Chem Commun.

[26] Hosoya M, Manaka A, Nishijima S, Tsuno N (2021). Asian J Org Chem.

[27] Qin J, Chen Q, Yang C, Huang Y (2016). J Alloys Compd.

[28] Okada A (2008). J Eur Ceram Soc.

[29] Wu G, Kuznetsov A V, Jasper W J (2011). J Aerosol Sci.

[30] Stratakis G A, Psarianos D L, Stamatelos A M (2002). Proc Inst Mech Eng, Part D.

[31] Mizukami T, Saito Y, Mase N (2020). Fine bubble generation device and method for generating fine bubbles. WO Patent.

[32] Mizukami T, Saito Y, Mase N (2020). Reaction device and reaction method using fine bubbles. WO Patent.

[33] Polterauer D, Roberge D M, Hanselmann P, Elsner P, Hone C A, Kappe C O (2021). React Chem Eng.

[34] Venkateshwarlu G, Chandravadana M V, Pandey M, Tewari R P, Selvaraj Y (2000). Flavour Fragrance J.

[35] Lin Y, Huang R, Sun X, Yu X, Xiao Y, Wang L, Hu W, Zhong T (2021). Food Control.

[36] Wang J, Zhou R-G, Wu T, Yang T, Qin Q-X, Li I, Yang B, Yang J (2012). J Chem Res.

[37] Chen Y, Zhang X, Wang X, Drout R J, Mian M R, Cao R, Ma K, Xia Q, Li Z, Farha O K (2021). J Am Chem Soc.

[38] Steves J E, Stahl S S (2013). J Am Chem Soc.

[39] Hayashi M, Sasano Y, Nagasawa S, Shibuya M, Iwabuchi Y (2011). Chem Pharm Bull.

[40] Hong M, Min J, Wu S, Cui H, Zhao Y, Li J, Wang S (2019). ACS Omega.

[41] (2023). ICH website.

[42] Nishimura T, Onoue T, Ohe K, Uemura S (1998). Tetrahedron Lett.

[43] Liu W, Twilton J, Wei B, Lee M, Hopkins M N, Bacsa J, Stahl S S, Davies H M L (2021). ACS Catal.

[44] Ji H-B, Wang T-T, Zhang M-Y, Chen Q-L, Gao X-N (2007). React Kinet Catal Lett.

[45] Wang L, Li J, Yang H, Lv Y, Gao S (2012). J Org Chem.

[46] Gao B, Zhang D, Li Y (2017). Eur J Inorg Chem.

[47] Nutting J E, Mao K, Stahl S S (2021). J Am Chem Soc.

[48] Hosoya M, Saito Y, Horiuchi Y (2023). ChemRxiv.

