# Supplementary material for: Honeycomb reactor: a promising device for streamlining aerobic oxidation under continuous-flow conditions
Source: Beilstein J Org Chem. 2023 May 31;19:752–63. doi: 10.3762/bjoc.19.55 (PMC10241100; doi:10.3762/bjoc.19.55)
Supplement: File 1 — Experimental procedures, supplementary experiments and NMR spectra. [file Beilstein_J_Org_Chem-19-752-s001.pdf]

BEILSTEIN JOURNAL OF ORGANIC CHEMISTRY

Supporting Information

for

Honeycomb reactor: a promising device for streamlining aerobic oxidation under continuous-flow conditions

Masahiro Hosoya, Yusuke Saito and Yousuke Horiuchi

Beilstein J. Org. Chem. **2023,** *19,* 752–763. doi:10.3762/bjoc.19.55

Experimental procedures, supplementary experiments and NMR spectra

License and Terms: This is a supporting information file under the terms of the Creative Commons Attribution License (https://creativecommons.org/licenses/by/4.0). Please note that the reuse, redistribution and reproduction in particular requires that the author(s) and source are credited and that individual graphics may be subject to special legal provisions.
The license is subject to the *Beilstein Journal of Organic Chemistry* terms and conditions: (https://www.beilstein-journals.org/bjoc/terms)

Contents:

S2–10: Experimental procedures
S11–12: Supplementary experiment: residence time distribution in the honeycomb reactor
S13: Supplementary experiment: evaluation of the reaction rate using the honeycomb reactor without BPR
S14: Supplementary experiment: evaluation of the transition of reaction rate using the honeycomb reactor
S15: HPLC method for aerobic oxidation
S16: ^{1}H NMR and ^{13}C NMR spectra of **2a**
S17: Detailed description of the setup for the honeycomb reactor
S18: Author contributions

Experimental procedures

General information

Solvents and reagents were purchased from commercial sources and used without further purification. Benzyl alcohols (**1a–h**) and authentic samples (**2a**, **3a** and **2b–h**) were purchased from Tokyo Chemical Industry Co., Ltd. TEMPO was purchased from Tokyo Chemical Industry Co., Ltd. and $Fe(NO_3)_3{\cdot}9H_2O$ was purchased from FUJIFILM Wako Pure Chemical Corporation. High performance liquid chromatographic (HPLC) analysis was carried out using a Shimadzu LC-2010CHT. ^1H NMR and ^{13}C NMR were recorded on a Bruker AVANCE III HD 400 MHz. A batch reaction was conducted using EYELA ChemiStation PPS-1511 with a cross-shaped stirring bar unless otherwise noted. The heat of reaction was evaluated using a Mettler Toledo EasyMax 102 (100 mL). IR spectra were measured using Mettler Toledo ReactIR 702L with a TE-MCT detector connected with a flow cell (DS dicomp micro flow cell, internal volume: 50μL). Differential Scanning Calorimetry (DSC) was carried out using a Mettler Toledo Thermal Analysis System DSC 3+.

A standard tube reactor was purchased from Vapourtec Ltd (Vapourtec standard coiled tubular reactor, inner diameter: 1.0 mm, internal volume: 20 mL (10 mL × 2)). A tube reactor equipped with a static mixer was purchased from Vapourtec Ltd (large diameter tubular reactor for rapid mixing, inner diameter: 3.2 mm, internal volume: 20 mL). A T-shape mixer was purchased from EYELA Co., Ltd. (JTF-310, through hole 1.0 mm). A backpressure regulator (BPR) was purchased from DFC Co. Ltd (FC-BPV1-250). The Vapourtec V-3 is a peristaltic pump. Mass flow controller (MFC) was purchased from Bronkhorst High-Tech B.V. (FG-201CV-RGD-11-E-DA-000, the indicated volume of O_2 means the volume at the standard conditions (273K, 1 atm), not the observed volume).

Flow rates were calibrated manually as follows: the weight of the fed amount was measured for 1 min using AcOH and the measured weight was converted to the volume using the density (pump A). The volume of the fed O_2 gas was collected for 1 min over water, and the measured volume was converted to that under standard conditions (273 K, 1 atm) according to Boyle–Charles law (MFC).

Detailed information for the honeycomb reactor (see also Figure 1)

Porous material: Cordierite ($2MgO \cdot 2Al_2O_3 \cdot 5SiO_2$)

Average pore diameter of the porous material: 12 μm

Porosity of the porous material: 48%

Outer dimensions of the honeycomb reactor: Φ30 × 50 mm

Cell number of the honeycomb reactor: 300 cells/in^2

Size of each cell: 1.14 mm on a side (cross section of each cell: square)

Thickness of the porous walls: 300 μm

Internal volume: 25 mL

The honeycomb reactor was coated with fluorine-based film for leak prevention (cf. WO2022024748, February 3, 2022.) and was contained in a housing made of stainless steel (see also Figure 3c).

[Cleaning method]

After the use of the honeycomb reactor, the reaction solvent was fed under 8 bar until the remaining O_2 gas was not observed. After feeding the reaction solvent, the BPR was eliminated, and the honeycomb reactor was flushed with inert N_2 gas until the reaction solvent was not observed.

Representative batch procedure for reaction screening (entry 3 in Table 1)

4-Methoxybenzyl alcohol (**1a**, 138.1 mg, 1.00 mmol), TEMPO (7.8 mg, 0.05 mmol, 0.05 equiv), $Fe(NO_3)_3 \cdot 9H_2O$ (20.1 mg, 0.05 mmol, 0.05 equiv) and AcOH (1 mL, 7 mL/g) were mixed. The reaction solution was vigorously stirred at 25 °C in open air. The reaction solution (10 μL) was sampled and diluted in $MeCN/H_2O$ (80/20 (v/v), 1.5 mL). The diluted sample was analyzed by HPLC.

Representative batch procedure for reaction optimization (entry 6 in Table 2)

(Reaction monitoring and calculation of quantitative yield)

4-Methoxybenzyl alcohol (**1a**, 345.3 mg, 2.50 mmol), TEMPO (7.8 mg, 0.05 mmol, 0.02 equiv), $Fe(NO_3)_3 \cdot 9H_2O$ (20.2 mg, 0.05 mmol, 0.02 equiv) and AcOH (1 mL, 3 mL/g) were mixed. The reaction solution was vigorously stirred at 80 °C under an O_2 balloon. The reaction solution (10 μL) was sampled and diluted in $MeCN/H_2O$ (80/20 (v/v), 1 mL). The diluted sample was further diluted 4 times with $MeCN/H_2O$ (80/20 (v/v)) and analyzed by HPLC. (In the calculation of quantitative yield: The reaction solution (200 mg) was sampled and diluted in a measuring flask (100 mL) with $MeCN/H_2O$ (80/20 (v/v)) The quantitative yield was calculated using the authentic sample **2a** on HPLC.

(Calculation of isolated yield)

4-Methoxybenzyl alcohol (**1a**, 500.9 mg, 3.63 mmol), TEMPO (11.3 mg, 0.07 mmol, 0.02 equiv), $Fe(NO_3)_3 \cdot 9H_2O$ (29.2 mg, 0.07 mmol, 0.02 equiv) and AcOH (1.5 mL, 3 mL/g) were mixed. The reaction solution was vigorously stirred at 80 °C under an O_2 balloon for 20 min. After the completion of reaction confirmed by HPLC, the reaction solution was quenched with AcOEt (20 mL) / 7.5% Na_2CO_3 aq (20 mL). The aqueous phase was extracted with AcOEt (20 mL). The combined organic phases were washed with water (10 mL) and evaporated, and the residue was purified by the flash column chromatography (*n*-heptane/AcOEt = 9/1) to give *p*-anisaldehyde (**2a**) as colorless oil (462.7 mg, 94%).

^1H NMR (400 MHz, $CDCl_3$) δ 9.89 (1H, s), 7.84 (2H, d, J = 8.8 Hz), 7.01 (2H, d, J = 8.8 Hz), 3.89 (3H, s).

^{13}C NMR (100 MHz, $CDCl_3$) δ 190.90, 164.76, 132.11, 130.15, 114.46, 55.71.

These NMR spectra were consistent with the previously reported spectra (*Tetrahedron* **2013**, *69*, 9192–9199.).

Evaluation of the heat of reaction (Figure 2)

TEMPO (180.9 mg, 1.16 mmol, 0.02 equiv), $Fe(NO_3)_3 \cdot 9H_2O$ (468.0 mg, 1.16 mmol, 0.02 equiv) and AcOH (24.18 g) were mixed in EasyMax 102 (100 mL). The catalyst solution was heated to 80 °C under an O_2 balloon. 4-Methoxybenzyl alcohol (**1a**) (8.00 g, 57.90 mmol) was added to the catalyst solution for 3 min, and the vessel of **1a** was washed with AcOH (1.00 g) (total amount of AcOH: 25.18 g, 24 mL, 3 mL/g), which was added to the reaction solution. The reaction solution was vigorously stirred at 80°C under the O_2 balloon until the exotherm was not detected in EasyMax. After the exotherm was not detected, the reaction solution (10 µL) was sampled and diluted in $MeCN/H_2O$ (80/20 (v/v), 1 mL). The diluted sample was further diluted 4 times with $MeCN/H_2O$ (80/20 (v/v)) and analyzed by HPLC, which confirmed the completion of reaction.

The time course of $T_r - T_j$ was shown in Figure S1, and ΔH_R *and* ΔT_{ad} were calculated from the following formulae. ΔH_R was calculated to be 161 kJ/mol, and ΔT_{ad} was calculated to be 138 K (= 161 × 0.05790 / (0.03383 × 2.0)).

$$q = U \times A \times (T_r - T_j)$$

$$\Delta H_R = \frac{\int_{T_0}^{T_1} q dt}{M}$$

$$\Delta T_{ad} = \frac{\Delta H_R \times M}{W \times C_p}$$

q: Amount of heat transfer [J/s]

U: Overall heat transfer coefficient [J/m^2·K·s] (188 J/m^2·K·s)

A: Heat transfer area [m^2] (0.00422 m^2)

T_r: Internal temperature [K] (See Figure S1)

T_j: Jacket temperature [K] (See Figure S1)

ΔH_R: Heat of reaction [kJ/mol]

T_0: Time at the start of reaction [s]

T_1: Time at the end of reaction [s]

t: Reaction time [s]

M: Input amount of starting material **1a** [mol] (0.05790 mol)

ΔT_{ad}: Adiabatic heating [K]

W: Reaction mass [kg] (0.03383 kg)

C_p: Specific heat [kJ/kg·K] (2.0 kJ/kg·K)

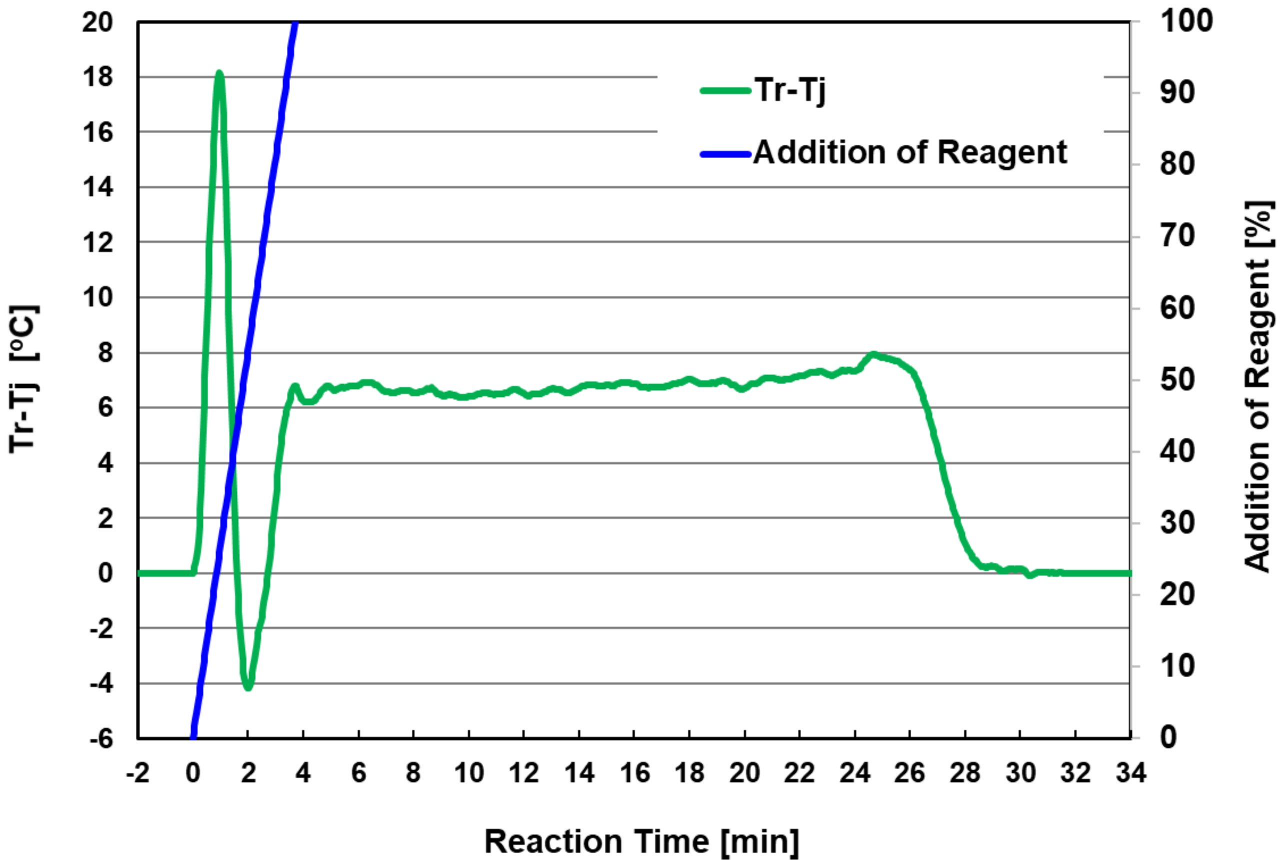

Figure S1. Time course of $T_r - T_j$.

DSC analysis of the reaction solution did not show any exotherms (Table S1).

Table S1. Summary of DSC Data.

Sample Name [a]	Process Temp (°C)	T_{onset} (°C)	Enthalpy (J/g)
4-Methoxybenzyl alcohol	–	–	–
TEMPO	–	37	−40 (melting point)
$Fe(NO_3)_3{\cdot}9H_2O$	–	54	−209 (melting point)
		178	214
Reaction Solution	80	–	–

a) Measured temperature range: 30–250 °C, thermal analysis crucibles made of stainless steel, 10.00 K/min, N_2 40.0 mL/min.

Flow procedures for evaluating the reaction rate (Table 3)

(Entry 1)

The reaction solution was fed by the Vapourtec V-3, and O_2 gas was fed by MFC; the reaction mixture was a solution of 4-methoxybenzyl alcohol (**1a**, 1.00 g, 7.24 mmol), TEMPO (22.6 mg, 0.14 mmol, 0.02 equiv) and $Fe(NO_3)_3 \cdot 9H_2O$ (58.5 mg, 0.14 mmol, 0.02 equiv) in AcOH (120 mL) (0.060 mmol/mL). Flow rate of the reaction solution was set as 0.50 mL/min, that of O_2 gas was set as 8.0 mL/min.

The equivalent of O_2 gas was calculated to be 12 equiv (excess amount).

The detailed calculation of the equivalent was shown as follows: feeding rate of **1a**: 0.060×0.50 = 0.030 mmol/min, feeding rate of O_2 gas: 8.0/22.4 = 0.357 mmol/min, 0.357/0.030 = 12.

The reaction solution and O_2 gas were mixed by a T-shape mixer (through hole: 1.0 mm), and the slug flow was passed through the standard tube reactor (internal volume: 20 mL) at 80°C for jacket temperature under 8 bar (atmospheric pressure: 1 bar).

After running for 30 min, the obtained reaction solution (100 μL) was sampled and diluted in $MeCN/H_2O$ (80/20 (v/v), 0.9 mL). The diluted sample was analyzed by HPLC.

(Entry 2)

The reaction solution was fed by a Vapourtec V-3, and O_2 gas was fed by MFC; the reaction mixture was a solution of 4-methoxybenzyl alcohol (**1a**, 2.00 g, 14.47 mmol), TEMPO (45.2 mg, 0.29 mmol, 0.02 equiv) and $Fe(NO_3)_3 \cdot 9H_2O$ (117.0 mg, 0.29 mmol, 0.02 equiv) in AcOH (240 mL) (0.060 mmol/mL). Flow rate of the reaction solution was set as 1.50 mL/min, that of O_2 gas was set as 24.0 mL/min.

The equivalent of O_2 gas in each cycle was calculated to be 12 equiv (excess amount).

The detailed calculation of the equivalent was shown as follows: feeding rate of **1a**: 0.060 × 1.50 = 0.090 mmol/min, feeding rate of O_2 gas: 24.0/22.4 = 1.071 mmol/min, 1.071/0.090 = 12.

The reaction solution and O_2 gas were mixed by a T-shape mixer (through hole: 1.0 mm), and the slug flow was passed through the standard tube reactor (internal volume: 20 mL) at 80 °C for jacket temperature under 8 bar (atmospheric pressure: 1 bar).

The obtained reaction solution was stored at 15 °C for 30 min (running time: 10–40 min) (the first cycle).

The stored reaction solution was fed in the similar manner to the first cycle (the second cycle). The obtained reaction solution was stored at 15 °C for 20 min (running time: 10–30 min).

The stored reaction solution was fed in the similar manner to the second cycle (the third cycle). After running 10 min, the obtained reaction solution (100 μL) was sampled and diluted in $MeCN/H_2O$ (80/20 (v/v), 0.9 mL). The diluted sample was analyzed by HPLC.

(Entry 3)

Entry 3 was conducted using the tube reactor with static mixer (internal volume: 20 mL) in the similar manner to entry 1 using the standard tube reactor.

After running for 50 min, the obtained reaction solution (100 µL) was sampled and diluted in MeCN/H_2O (80/20 (v/v), 0.9 mL). The diluted sample was analyzed by HPLC.

(Entry 4)

Entry 4 was conducted using the tube reactor with static mixer (internal volume: 20 mL) in the similar manner to entry 2 using the standard tube reactor.

The obtained reaction solution was stored at 15 °C for 45 min (running time: 15–60 min) (the first cycle). The stored reaction solution was fed in the similar manner to the first cycle (the second cycle). The obtained reaction solution was stored at 15 °C for 30 min (running time: 15–45 min).

The stored reaction solution was fed in the similar manner to the second cycle (the third cycle). After running 15 min, the obtained reaction solution (100 µL) was sampled and diluted in MeCN/H_2O (80/20 (v/v), 0.9 mL). The diluted sample was analyzed by HPLC.

(Entries 5 and 6)

Entries 5 and 6 were conducted using the honeycomb reactor (internal volume: 25 mL) in the similar manner to entry 1 using the standard tube reactor.

After running for 90 min, the obtained reaction solution (100 µL) was sampled and diluted in MeCN/H_2O (80/20 (v/v), 0.9 mL). The diluted sample was analyzed by HPLC.

(Entry 7)

Entry 7 was conducted using the honeycomb reactor (internal volume: 25 mL) in the similar manner to entry 2 using the standard tube reactor.

The obtained reaction solution was stored at 15°C for 70 min (running time: 30–100 min) (the first cycle). The stored reaction solution was fed in the similar manner to the first cycle (the second cycle). The obtained reaction solution was stored at 15°C for 40 min (running time: 30–70 min).

The stored reaction solution was fed in the similar manner to the second cycle (the third cycle). After running 30 min, the obtained reaction solution (100 µL) was sampled and diluted in MeCN/H_2O (80/20 (v/v), 0.9 mL). The diluted sample was analyzed by HPLC.

(Entry 8)

The reaction solution was fed by the Vapourtec V-3, and O_2 gas was fed by MFC; the reaction mixture was a solution of 4-methoxybenzyl alcohol (**1a**, 2.00g, 14.47 mmol), TEMPO (45.2 mg, 0.29 mmol, 0.02 equiv) and $Fe(NO_3)_3{\cdot}9H_2O$ (117.1 mg, 0.29 mmol, 0.02 equiv) in AcOH (240 mL) (0.060 mmol/mL).

The reaction solution and O_2 gas were mixed by a T-shape mixer (through hole: 1.0 mm), and the slug flow was passed through the three honeycomb reactors in series (internal volume: 75 mL) at 80 °C for jacket temperature under 8 bar (atmospheric pressure: 1 bar). After running for 90 min, the obtained reaction solution (100 μL) was sampled and diluted in MeCN/H_2O (80/20 (v/v), 0.9 mL). The diluted sample was analyzed by HPLC.

Flow procedure for maximizing the throughput (Table 4)

The reaction solution was fed by the Vapourtec V-3, and O_2 gas was fed by MFC; the reaction mixture was a solution of 4-methoxybenzyl alcohol (**1a**, 60.00 g, 434.25 mmol), TEMPO (1.36 g, 8.70 mmol, 0.02 equiv) and $Fe(NO_3)_3{\cdot}9H_2O$ (3.51 g, 8.69 mmol, 0.02 equiv) in AcOH (180 mL) (the concentration of **1a** was calculated to be 1.83 mmol/mL). The flow rate of the reaction solution was set as 3.75 mL/min, that of O_2 gas was set as 60.0 mL/min.

The equivalent of O_2 in each cycle was calculated to be 0.4 equiv.

The detailed calculation of the equivalent was shown as follows: feeding rate of **1a**: $1.83 \times 3.75 = 6.863$ mmol/min, feeding rate of O_2 gas: $60.0/22.4 = 2.679$ mmol/min, $2.679/6.863 = 0.4$.

The reaction solution and O_2 gas were mixed by a T-shape mixer (through hole: 1.0 mm), and the slug flow was passed through the honeycomb reactor (internal volume: 25 mL) at 80 °C for jacket temperature under 8 bar (atmospheric pressure: 1 bar).

The obtained reaction solution was stored at 15 °C for 35 min (running time: 15–50 min) (the first cycle). The stored reaction solution was fed in the similar manner to the first cycle (the second cycle). The obtained reaction solution was stored at 15 °C for 20 min (running time: 15–35 min). The stored reaction solution was fed in the similar manner to the second cycle (the third cycle). After running 15 min, the reaction solution (10 μL) was sampled and diluted in MeCN/H_2O (80/20 (v/v), 1 mL). The diluted sample was further diluted 4 times with MeCN/H_2O (80/20 (v/v)) and analyzed by HPLC. (In the calculation of quantitative yield: The reaction solution (200 mg) was sampled and diluted in a measuring flask (100 mL) with MeCN/H_2O (80/20 (v/v)) The quantitative yields were calculated using the authentic samples **2a** and **3a** on HPLC.

Flow procedures for substrate scope (Table 5)

The reaction solution was fed by the Vapourtec V-3, and O_2 gas was fed by MFC; the reaction mixture was a solution of benzyl alcohols (**1a–h**, 7.24 mmol), TEMPO (22.6 mg, 0.14 mmol, 0.02 equiv) and $Fe(NO_3)_3{\cdot}9H_2O$ (58.5 mg, 0.14 mmol, 0.02 equiv) in AcOH (120 mL) (0.060 mmol/mL). In the case of entries 9 and 10, $Cu(NO_3)_2{\cdot}3H_2O$ (52.5 mg, 0.22 mmol, 0.03 equiv) and $Zn(NO_3)_2{\cdot}6H_2O$ (64.6 mg, 0.22 mmol, 0.03 equiv) were used instead of $Fe(NO_3)_3{\cdot}9H_2O$ (0.02 equiv), respectively.

(Standard tube reactor)

Flow rate of the reaction solution was set as 0.50 mL/min, that of O_2 gas was set as 8.0 mL/min.

The reaction solution and O_2 gas were mixed by a T-shape mixer (through hole: 1.0 mm), and the slug flow was passed through the standard tube reactor (internal volume: 20 mL) at 80 °C for jacket temperature under 8 bar (atmospheric pressure: 1 bar).

After running for 30 min, the obtained reaction solution (200 mg) was sampled and diluted in a measuring flask (10 mL) with $MeCN/H_2O$ (80/20 (v/v)). The diluted sample was analyzed by HPLC. The quantitative yields were calculated using the authentic samples **2a–h** on HPLC.

(Honeycomb reactor)

Flow rate of the reaction solution was set as 0.63 mL/min, that of O_2 gas was set as 10.0 mL/min.

The reaction solution and O_2 gas were mixed by a T-shape mixer (through hole: 1.0 mm), and the slug flow was passed through the honeycomb reactor (internal volume: 25 mL) at 80 °C for jacket temperature under 8 bar (atmospheric pressure: 1 bar).

After running for 90 min, the obtained reaction solution (200 mg) was sampled and diluted in a measuring flask (10 mL) with $MeCN/H_2O$ (80/20 (v/v)). The diluted sample was analyzed by HPLC. The quantitative yields were calculated using the authentic samples **2a–h** on HPLC.

Supplementary experiment: residence time distribution in the honeycomb reactor

The sample solution was prepared by dissolving *p*-anisaldehyde (**2a**, 10.00 g) in AcOH 190.00 g. The sample solution was fed by the Vapourtec V-3, and the flow rate of the sample solution was set as 2.50 mL/min. The sample solution was passed through the honeycomb reactor (internal volume: 25 mL) at room temperature (25 °C) without back pressure or under 8 bar (atmospheric pressure: 1 bar) (residence time: 10.0 min). The flow cell connected with ReactIR was inserted just after the exit of the honeycomb reactor (see Scheme S1 for the flow setup). Before the sample solution was fed, the honeycomb reactor was filled with AcOH in advance. The start time of feeding the sample solution was set as 0 min. After running for 60 min, the flow channel was switched from the sample solution of **2a** to AcOH. AcOH was fed until **2a** was undetectable. The running time was corrected based on the dead volume in the reactor setup.

In the IR measurements, the peak height at 1602 cm^{-1} (height to two-point baseline: 1636 to 1538 cm^{-1}) was measured. The peak height of AcOH at 1602 cm^{-1} was subtracted from the measured peak height of **2a** for the zero-point adjustment. The measured curves obtained from the real time monitoring using ReactIR show the residence time distribution during the flow experiments. These curves were compared to the theoretical one in Figure S2. When the honeycomb reactor was set vertically under 8 bar, the measured curve shifted to the far right in Figure S2 (Figure S2c), which indicated that this setup could make the most of the internal volume in the honeycomb reactor.

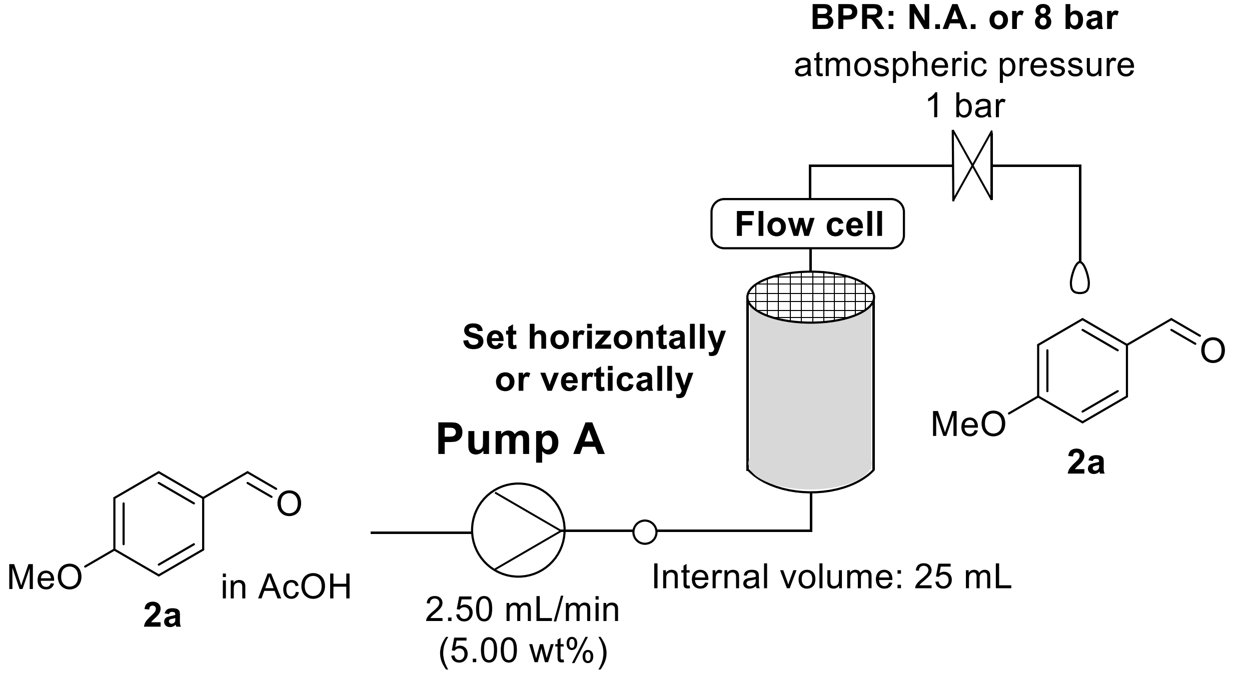

Scheme S1. Flow setup for investigating the residence time distribution.

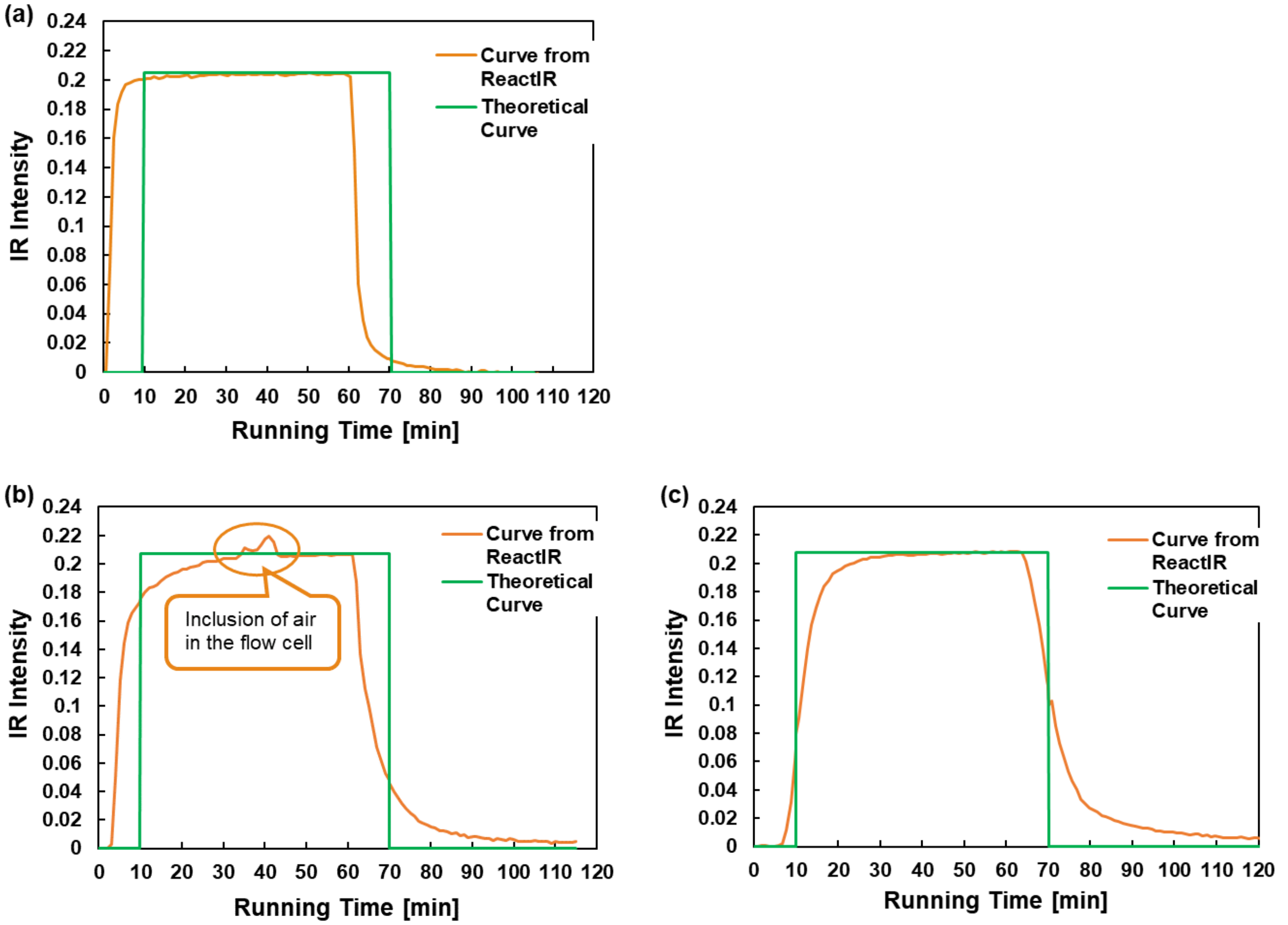

Figure S2. Residence time distribution using ReactIR in the honeycomb reactor: (a) Set horizontally without back pressure. (b) Set vertically without back pressure. (c) Set vertically under 8 bar.

Note that unexpected inclusion of air in the flow cell temporarily disturbed the IR measurement in (b), which was included in the reactor when the flow channel was switched from AcOH to solution of **2a**.

Supplementary experiment: evaluation of the reaction rate using the honeycomb reactor without BPR

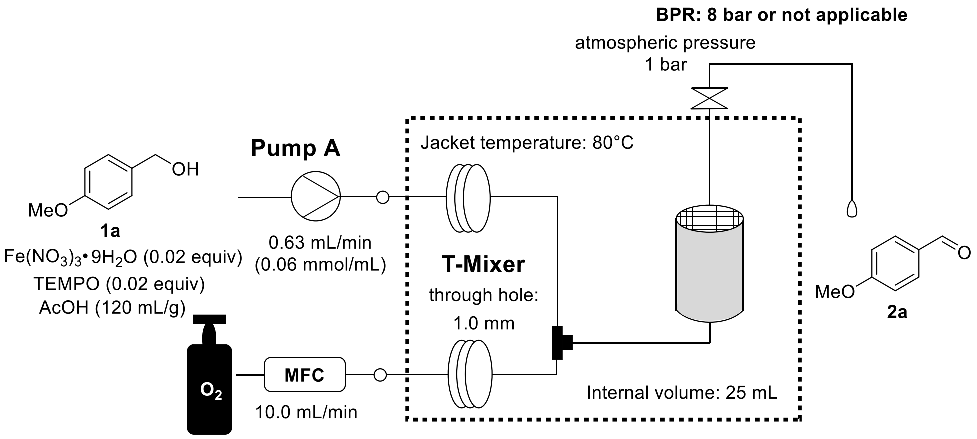

Scheme S2. Flow setup for evaluation of the reaction rate using the honeycomb reactor without BPR.

Table S2. Reaction rate using the honeycomb reactor without BPR.

Pump A / MFC (mL/min)	Internal volume (mL)	BPR	HPLC (area%)			Conv. (%) [a]
			**1a**	**2a**	**3a**	
0.63<br>10.0	25	8 bar	1.3	97.2	0.6	84
0.63<br>10.0	25	Not applicable	6.8	92.1	0.0	49

[a] Conv. (%) = **2a** (area%)/(**2a** (area%) + **1a** (area%)× 14.083) × 100. 14.083: relative sensitivity coefficient on HPLC (factor).

Without BPR, the residence time was shortened due to the increase of the observed volume of O_2 and the solubility of O_2 decreased, leading to the low conversion (Table S2).

Supplementary experiment: evaluation of the transition of reaction rate using the honeycomb reactor

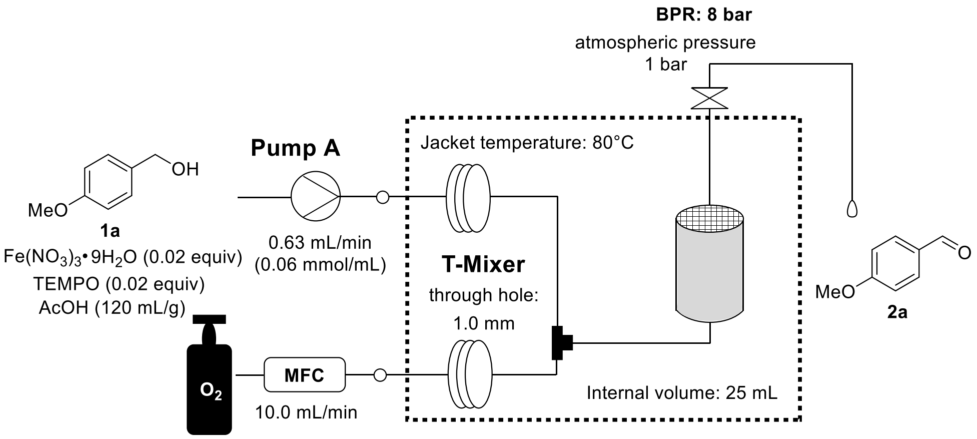

Scheme S3. Flow setup for evaluation of the transition of reaction rate using the honeycomb reactor.

Table S3. Transition of reaction rate using the honeycomb reactor.

Pump A / MFC (mL/min)	Internal volume (mL)	Running time (min)	HPLC (area%)			Conv. (%) [a]
			1a	2a	3a	
0.63	25	80	1.2	97.4	0.6	85
10.0		90	1.3	97.3	0.6	84
		100	1.3	97.2	0.6	84
		110	1.3	97.3	0.6	84
		120	1.3	97.2	0.6	84

[a] Conv. (%) = **2a** (area%)/(**2a** (area%) + **1a** (area%) × 14.083) × 100. 14.083: relative sensitivity coefficient on HPLC (factor).

No reduction in the conversion was observed for 120 min (Table S3).

HPLC method for aerobic oxidation

Column: Waters Xbridge C18, 4.6 × 150 mm, 5 µm

Mobile phase A: 0.3% TFA in purified water. Mobile phase B: MeCN.

B concentration (Gradient): 0.0–15.0 min 15–20%, 15.0–17.0 min 20–85%, 17.0–22.0 min 85%, 22.0–22.1 min 85–15%, 22.1–30.0 min 15%.

Flow rate: 1.0 mL/min

Injection volume: 5 µL

Column temperature: 40 °C

Wavelength: 254 nm

Sample was diluted with MeCN/H_2O (80/20 (v/v))

The relative sensitivity coefficients at 254 nm were determined to calculate Conv (%) and summarized in Table S4.

Table S4. Summary of relative sensitivity coefficients.

Item	Relative sensitivity coefficient
**1a/2a**	14.083
**1b/2b**	72.552
**1c/2c**	76.628
**1d/2d**	85.399
**1e/2e**	10.266
**1f/2f**	3.806
**1g/2g**	24.192
**1h/2h**	24.904

Compound **2a**; ^{1}H NMR and ^{13}C NMR spectra

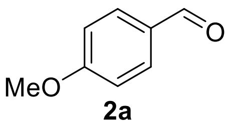

2a

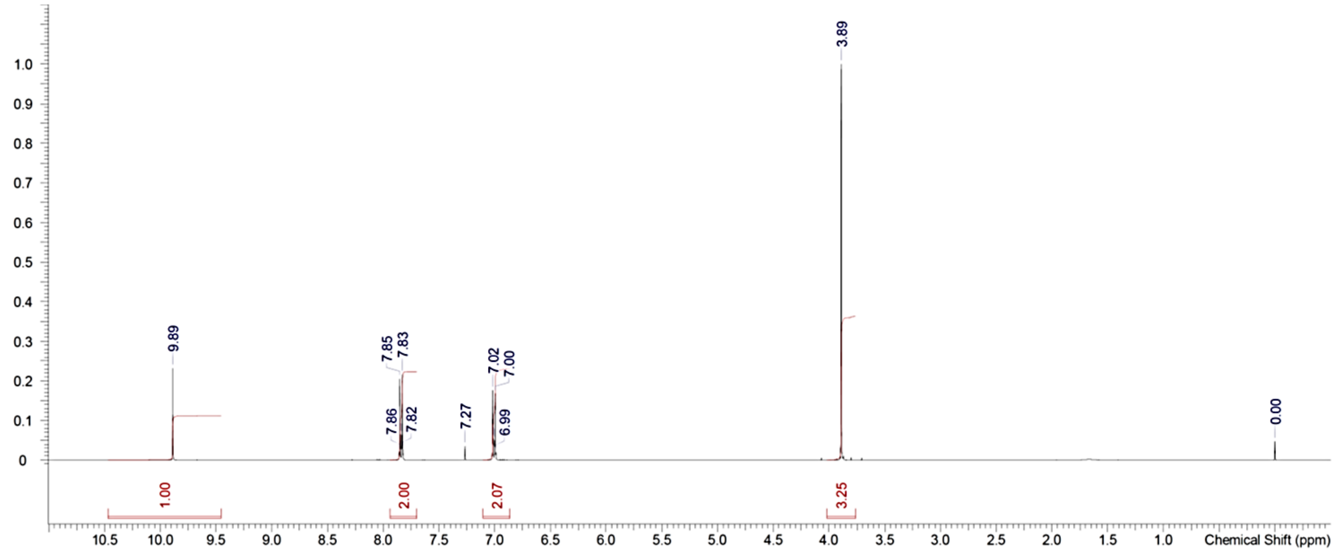

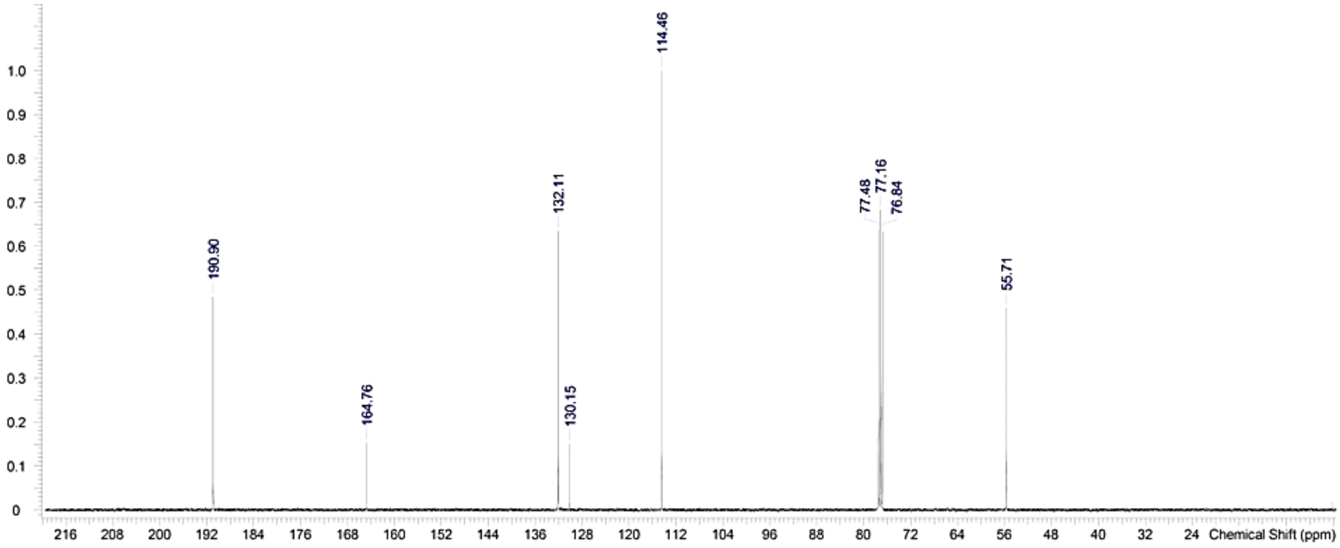

Detailed description of the setup for the honeycomb reactor

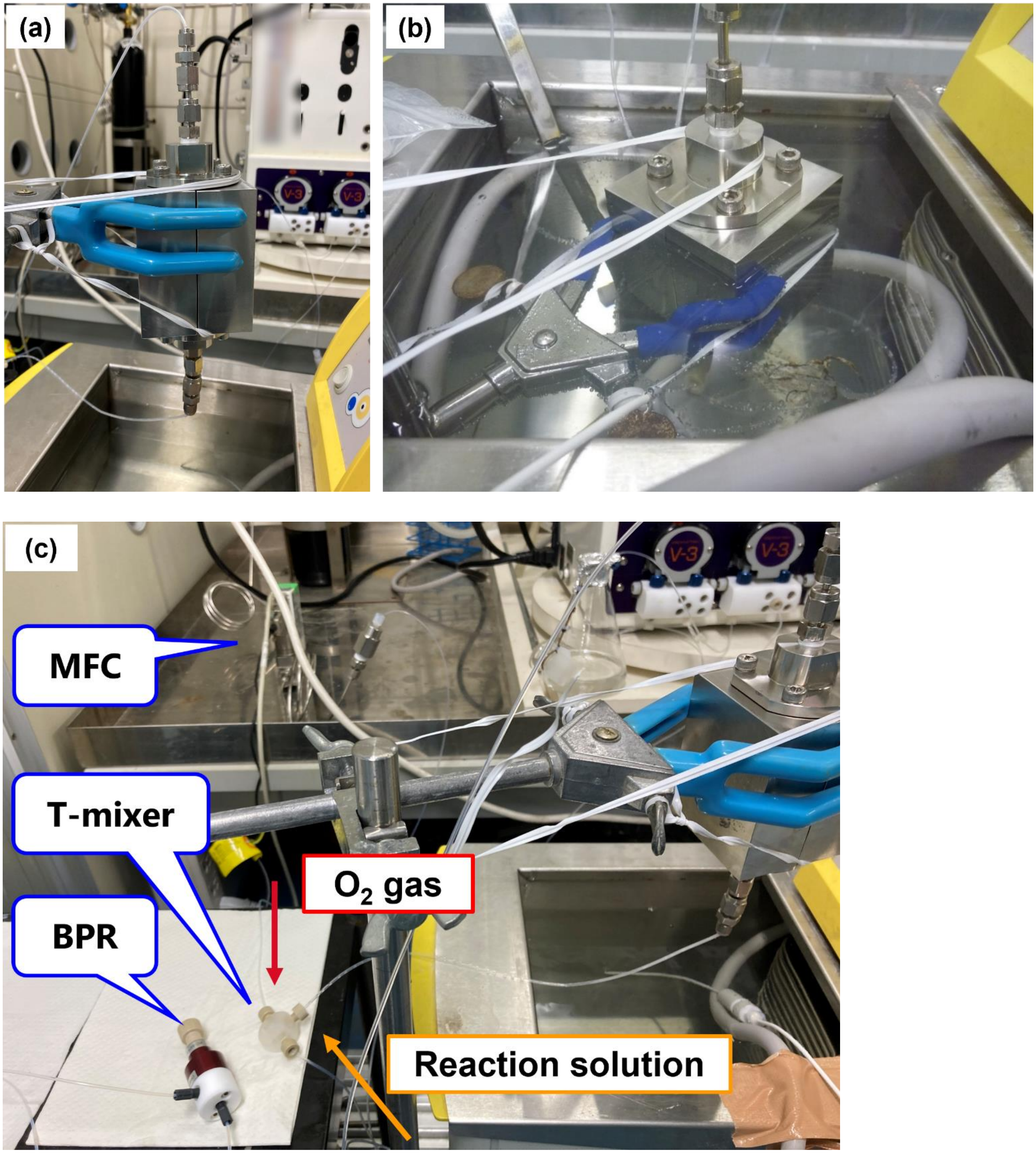

Figure S3. Detailed description of the setup for the honeycomb reactor. (a) Honeycomb reactor set vertically. (b) Immersed honeycomb reactor in water bath. (c) Overview.

Author contributions

This study was conceptualized and designed by Masahiro Hosoya. The design and assembly for the honeycomb reactor were performed by Yusuke Saito and Yousuke Horiuchi. Data collection and analysis were mainly performed by Masahiro Hosoya. The first draft of the manuscript was written by Masahiro Hosoya, and all the authors commented on the previous versions of the manuscript. All authors read and approved the final manuscript.
